# Need for transition medicine in pediatric surgery – health related quality of life in adolescents and young adults with congenital malformations

**DOI:** 10.1515/iss-2021-0019

**Published:** 2022-01-07

**Authors:** Marie Uecker, Benno Ure, Julia Hannah Quitmann, Jens Dingemann

**Affiliations:** Center of Pediatric Surgery, Hannover Medical School and Bult Children’s Hospital, Hannover, Germany; Department of Medical Psychology, University Medical Center Hamburg-Eppendorf, Hamburg, Germany

**Keywords:** anorectal malformations, biliary atresia, coledochal cyst, congenital diaphragmatic hernia, esophageal atresia, health-related quality of life, Hirschsprung’s disease, transition medicine

## Abstract

Survival rates of patients with visceral congenital malformations have increased considerably. However, long-term morbidity in these patients is high. In the last decades, these circumstances have led to a shift in goals of caretakers and researchers with a new focus on patients’ perspectives and long-term morbidity. Health-related quality of life (HrQoL) is the most commonly used patient-reported outcome measure to assess the impact of chronic symptoms on patients’ everyday lives. Most pediatric surgical conditions can cause a significantly decreased HrQoL in affected patients compared to the healthy population. In order to guarantee life-long care and to minimize the impact on HrQoL a regular interdisciplinary follow-up is obligatory. The period of transition from child-centered to adult-oriented medicine represents a critical phase in the long-term care of these complex patients. This scoping review aims to summarize relevant pediatric surgical conditions focusing on long-term-morbidity and HrQoL assessment in order to demonstrate the necessity for a well-structured and standardized transition for pediatric surgical patients.

## Introduction

Pediatric surgery provides operative solutions for patients born with congenital malformations. For a long time, the investigation of outcomes has been limited to clinical and functional parameters of the immediate postoperative period. Improvement of neonatal and perioperative medicine as well as follow-up care has led to increased survival rates in these patients with the vast majority surviving into adulthood. The decrease in mortality has shifted the focus of pediatric surgical care and research to the impact of major postoperative sequelae and long-term morbidity on patients’ lives [1]. The extent of these long-term morbidities varies considerably. While some patients move on to lead a normal life after successful initial surgery, others struggle with chronic symptoms as well as physical and psychological impairments.

As these factors often majorly impact patients’ well-being and everyday life, the evaluation of patients’ perceptions regarding their disease represents an important area of interest for pediatric surgical outcome analysis and research. The inclusion of the patient’s perspective in health research has been termed “patient-reported outcome measures” (PROMs) with health-related quality of life (HrQoL) being considered one of the most important and best validated PROMs [2].

**Figure 1:**
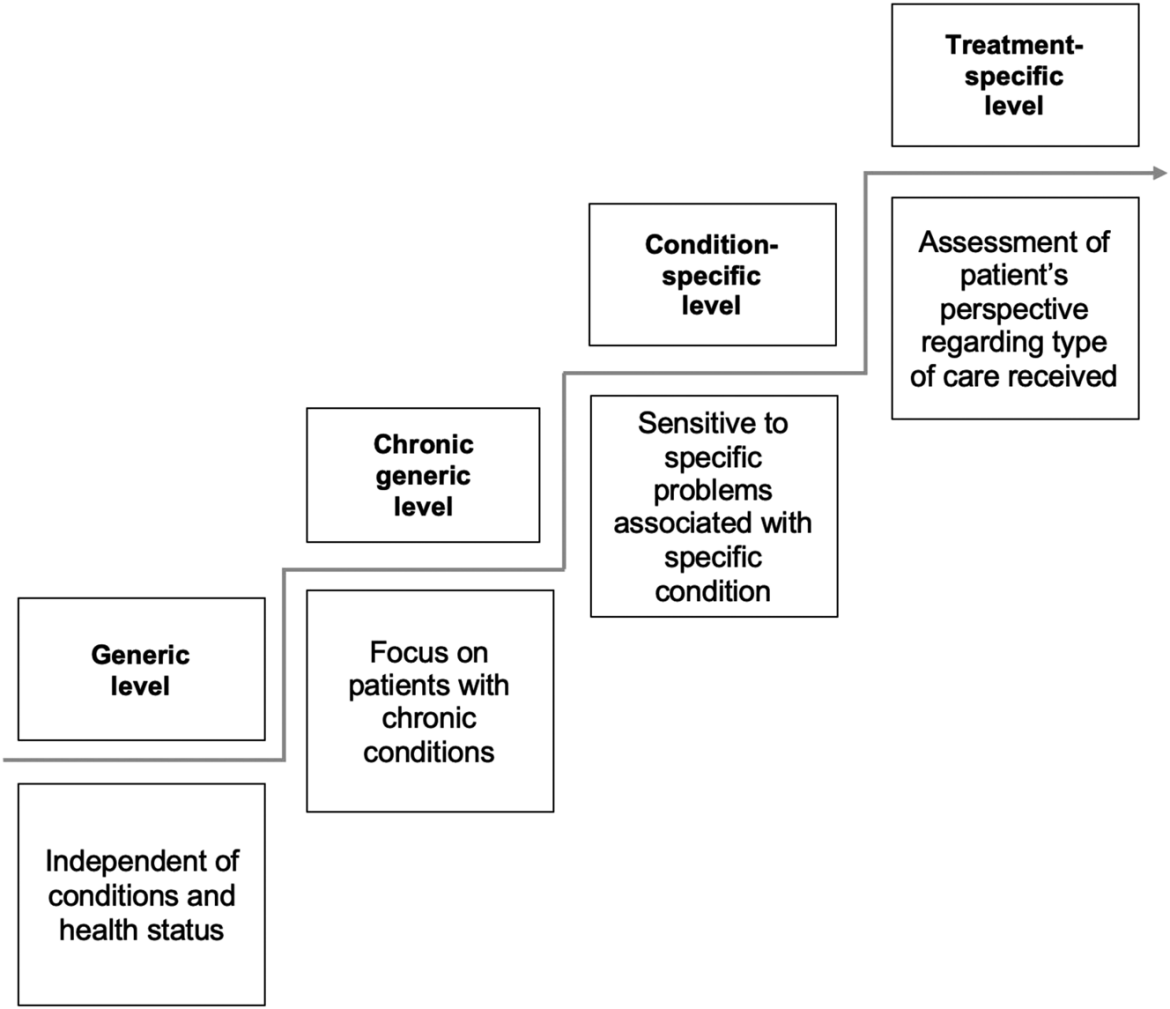
Levels of instruments to measure HrQoL [2].

The World Health Organization (WHO) has defined health as physical, mental and social well-being [3]. The assessment of HrQoL aims at measuring the subjective perception of health, based on how a disease affects the patients’ daily and social life as well as their physical and mental well-being [2]. HrQoL can be measured using four different types of instruments: generic, chronic-generic, condition-specific and treatment-specific instruments (Figure 1) [4]. Generic questionnaires represent the full range of health conditions, address groups independent of their respective health state and are effective for comparisons between two cohorts (e.g. patients with esophageal atresia and healthy controls). Chronic-generic instruments are focusing on a chronic condition independent of its specific characteristics, while specific questionnaires are tailored to problems associated with a specific condition (e.g. anorectal malformation) or symptom (e.g. incontinence) or treatment (e.g. patients receiving a liver transplantation).

The amount of both generic HrQoL instruments for children of different ages and their families as well as disease-specific instruments targeting the field of pediatric surgery has increased in recent years [2]. However, the range of available instruments elevates the risk for incorrect application and interpretation leading to heterogenous data that may additionally be biased by subjective interests of the researcher [5]. A careful choice of validated instruments is required in order to guarantee a comprehensive understanding and objective outcome evaluation of HrQoL [6].

Evaluations of therapeutic interventions benefit from the conjoint use of generic and condition-specific assessment measures, since generic measures facilitate cross-condition comparisons, while disease-specific instruments will allow more detailed and reliable results as they are tailored to the individual condition [2]. Still, disease-specific instruments have been implemented for only a limited number of pediatric surgical conditions (e.g. esophageal atresia [7], anorectal malformations/Hirschsprung’s disease [8]) (Table 2).

**Table 1:** Long-term morbidity of important visceral pediatric surgical conditions.

Esophageal atresia [9]	Anorectal malformations and Hirschsprung’s disease [24]	Short bowel syndrome [37, 38]	Biliary atresia and congenital choledochal malformation [46, 49, 63, 64]	Congenital diaphragmatic hernia [52, 53, 65]
– Dysphagia/dysmotility (21–84%) – Anastomotic stricture (40%) – Respiratory symptoms/tracheomalacia (30–69%) – Gastroesophageal reflux disease (46–76%) – Scoliosis (50% after open repair)	– Constipation (22–87%) – Stool incontinence/soiling (17–77%) – Urine incontinence (7–22%) – Sexual dysfunction (50%)	– Failure to thrive (53–72%) – Catheter related complications (0.41–1.5 episodes of infection/patient/year) – PN-associated liver disease (22–63%)	BA: – Portal hypertension (54–62%) – Recurring cholangitis (66–79%) – Terminal liver failure and liver transplantation (51–77%) CM: – Risk of malignancy (6–30%)	– Pulmonary impairment (25–50%) – Neurocognitive delay (40–57%) – Impaired cardiac function (25–45%) – Gastrointestinal symptoms (20–72%) – Musculosceletal sequelae (5–10%)

PN, parenteral nutrition; BA, biliary atresia; CM, choledochal malformation.

**Table 2:** Instruments used for assessment of pediatric surgical patients in studies targeting HrQoL.

	Esophageal atresia [7, 9]	Anorectal malformations and Hirschsprung’s disease [66]	Short bowel syndrome [37, 67]	Biliary atresia and congenital choledochal malformation [42, 68]	Congenital diaphragmatic hernia [57]
**Generic**	– PedsQL – SF-36 – DISABKIDS (chronic generic)	– PedsQL – CHQ – WHOQOL – KIDSCREEN – VSP-A – PedsQL – SF-36	– PedsQL	– PedsQL – CHQ – WHOQOL-BREF – SF36	– KIDSCREEN – PedsQL – ComQol – Family impact survey
**Symptom specific/treatment specific**	– GIQLI – GSRS – SDQ	– GIQLI – BFS – FIC QOL – PICS	– GIQLI – HPN-QOL	– PeLTQL – LTDS	n/a
**Disease specific**	– EA-QOL	– HAQL	– SBS-QoL *(validated for adults)*	n/a	n/a

PedsQL, pediatric quality of life inventory; SF-36, 36-item short form survey; GIQLI, gastrointestinal quality of life index; GSRS, gastrointestinal symptom rating scale; SDQ, swallowing disturbance questionnaire; EA-QOL, esophageal-atresia-quality-of-life-questionnaire; CHQ, child health questionnaire; WHOQOL, World Health Organization quality of life; VSP-A, Vécu et Santé Percue des Adolescents; BFS, bowel function score; FIC QOL, fecal incontinence quality of life; PICS, pediatric incontinence and constipation score; HAQL, Hirschsprung’s disease/anorectal malformation quality of life questionnaire; HNP-QOL, home parenteral nutrition-quality of life; SBS-QoL, short bowel syndrome-quality of life; PeLTQL, pediatric liver transplant quality of life; LTDS, liver transplant disability scale; ComQol, comprehensive quality of life scale; n/a, not available.

Chronic symptoms affect pediatric surgical patients for long periods of their lives and most conditions have a significant impact on patients’ HrQoL which is often decreased compared to healthy individuals [9]. The increased awareness of these factors has opened up the relatively young field of transition medicine, which targets the “transfer” of patients from pediatric to adult care. The transition of pediatric surgical patients poses a challenge due to the relatively rare conditions often not well known to adult practitioners. Patients previously treated by pediatric surgeons that were able to address most of their symptoms are transitioned into the much more specialized and organ specific care of adult medicine, often requiring multiple specialists to be able to tend to all their needs. A smooth transition ideally not only includes administrative and organizational efforts but also most importantly educates both the patient and the adult practitioner on the underlying disease.

About 40% of pediatric patients may be lost to follow-up during transition to adult medicine with transition programs having been shown to reduce this number to 10% [10]. Pediatric surgical patients without regular follow-up and organized care in adulthood are at risk to present with exacerbations of chronic symptoms that are potentially avoidable. A well-organized transition program not only positively affects patients’ long-term care and compliance but also prevents increased costs for the health system through early detection of relevant problems. The evaluation of such programs should include the assessment of the subjective impact using patient reported outcomes measures such as health-related quality of life.

Thanks to optimized care concepts, an increasing number of pediatric surgical patients now reach adulthood making transitional medicine an important aspect of pediatric surgery. This article aims to summarize and highlight the long-term morbidities and their effect on HrQoL investigating the current state of these aspects in relevant visceral pediatric surgical conditions in order to provide information for optimal transition of care for these complex patients.

### Esophageal atresia

Esophageal atresia (EA) is a congenital discontinuity of the esophagus with or without a tracheoesophageal fistula. Its incidence is estimated at 2.4/10,000 births [11]. The surgical approach depends on the distance between the two esophageal ends but if possible, primary anastomosis is preferred over esophageal replacements, e.g. gastric pull-up surgery. Neonatal morbidity and mortality are closely associated with birth weight and comorbidities, which exist in approximately 50% of patients (e.g. congenital heart disease, renal dysfunction, VACTERL association) [12].

The most frequent long-term morbidities include dysphagia/esophageal dysmotility (21–84%), anastomotic stricture (40%), tracheomalacia and respiratory symptoms, e.g. recurrent airway infections (30–69%) and gastroesophageal reflux (46–76%) with an increased risk of developing Barrett’s esophagus and carcinoma (Table 1) [13], [14], [15], [16]. Additionally, patients may suffer from psychological and physical (e.g. scoliosis) impairments caused by surgical scars [17]. The assessment of HrQoL in patients with EA is facilitated by the disease-specific EA-QOL-questionnaire that has been shown to improve outcome evaluation for patients with EA (Table 2) [7]. An uncomplicated operative course often leads to an excellent HrQoL after primary anastomosis, however most patients struggle with long-term health problems and concomitant anomalies that affect their HrQoL [18, 19]. Especially complicated courses of EA have been shown to significantly decrease HrQoL in affected patients [20, 21].

The well-known long-term health implications of EA patients and their impact on HrQoL stress the need of a systematic transition medicine for affected patients in order to maintain a disease-specific follow-up. Dingemann et al. observed a beneficial effect on both patients’ satisfaction as well as disease-specific knowledge in a comparative study subjecting adolescents with history of EA to a transition specific education program [22]. The results demonstrate the importance of patients’ education as a crucial part of successful transition. With approximately half of the EA patients suffering from concomitant anomalies of different organ systems, ensuring access to various specialists during transition to adult medicine is essential for these complex patients (Table 3).

**Table 3:** Important adult medical specialties required for transition of pediatric surgical patients.

Esophageal atresia	Anorectal malformations and Hirschsprung’s disease	Short bowel syndrome	Biliary atresia and congenital choledochal malformation	Congenital diaphragmatic hernia
– Gastroenterology – General surgery – Pulmonology – ENT – Orthopedics – Dietitians	– Gastroenterology – General surgery – Urology – Gynecology – Reproductive medicine	– Gastroenterology/hepatology – General surgery – Vascular surgery – Transplantation medicine – Dietitians	– Gastroenterology/hepatology (BA + CM) – General surgery (BA) – Transplantation medicine (BA)	– Pulmonology – Cardiology – General/thoracic surgery – Orthopedics

ENT, ear, nose and throat medicine; BA, biliary atresia; CM, choledochal malformation.

### Anorectal malformation and Hirschsprung’s disease

Anorectal malformation (ARM) and Hirschsprung’s disease (HD) are congenital conditions affecting the colon and/or rectum. Their incidence is estimated at 1.0–1.5 in 5,000 births [23]. ARM patients present with imperforate anus and frequently a fistula connected to the urogenital tract. The posterior sagittal anorectoplasty as the standard surgical approach aims at an anatomical reconstruction of the anus and resection of the fistula while preserving continence and sexual function. HD patients suffer from a congenital segmental aganglionosis leading to insufficient peristalsis. The standard surgical procedure for HD is resection of the aganglionotic bowel segment via different types of “pull-through operations” [24]. Despite successful surgical correction of the malformation, patients with ARM and HD regularly continue to face motility and continence issues that persist into adolescence and adulthood (Table 1). The majority of patients struggle with constipation (22–87%) and/or stool incontinence and soiling (17–77%) [25]. While urinary continence and sexual function in HD are most often impaired due to iatrogenic injury, in ARM patients these factors are mostly determined by severity of the disease which may include a hypoplastic pelvic floor or sacral agenesis [24]. Additionally, ARM patients frequently suffer from concomitant anomalies which are often VACTERL-associated [24]. Over 50% of patients with ARM experience sexual dysfunction with men most often affected after presence of a rectourethral fistula and women after cloacal malformation [26]. The latter group is also at risk for obstetrical complications such as premature births and complicated vaginal delivery with damage to the weak pelvic floor [27]. All of these long-term morbidities lead to impaired HrQoL in affected patients compared to healthy adolescents, with women more severely affected than men in both ARM and HD [28, 29]. The assessment of HrQoL of patients with ARM/HD is facilitated by the existence of the disease-specific HAQL (Hirschsprung’s disease/anorectal malformation quality of life questionnaire) as well as multiple available symptom-specific instruments (Table 2).

Cairo et al. found that structured transition medicine of patients with ARM and HD in the US is virtually non-existent even though it is considered highly necessary regarding the short- and long-term physical and psychosocial implications of the disease [30]. The lack of systematic and organized transition is confirmed by an international survey conducted by Giuliani et al. [31] However, awareness of the problem seems to increase and slight improvements in transitional care have been observed [32, 33].

Due to the diversity of symptoms and multiple affected organ systems, transition medicine for patients with ARM and HD should be addressed with a multidisciplinary team including gynecology, urology, gastroenterology as well as psychology (Table 3). In the presence of concomitant anomalies even more specialties are required to participate in the onward care.

### Short bowel syndrome

Short bowel syndrome (SBS) is defined by a loss of approximately 75% of the physiological length of the small bowel, in children most often as a result of early excessive damage (e.g. necrotizing enterocolitis, volvulus) or congenital malformations (e.g. abdominal wall defects, intestinal atresia). This leads to a severely decreased absorption of vital nutrients, fluids and electrolytes with patients being dependent on central line catheters and parenteral nutrition [34]. Surgical approaches such as Bianchi-lengthening procedure or serial transverse enteroplasty (STEP-procedure) aim to increase mucosal surface and absorption [35]. While in some patients dependency on parenteral nutrition decreases with growth and age, a bowel transplantation can serve as a last resort for others. Long-term survival rate of patients with SBS is up to 90% due to improved parenteral nutrition (PN), however, most patients continue to face relevant risks of catheter-related complications, such as infections or thrombosis as well as drastic failure to thrive and PN-associated liver disease (Table 1) [36]. HrQoL in pediatric patients with SBS has not been extensively studied but appears to be heavily affected [37, 38]. Appropriate instruments for pediatric HrQoL are lacking. A condition specific instrument is only available for the use in adult patients (Table 2).

The life-long necessary therapy and its potential for complications stress the need for a careful and thorough transition of patients. Data on transition for pediatric patients with SBS have not been published. However, transition is facilitated by the fact that SBS is a disease well known in adult medicine, making it easier to find adept gastroenterological teams to attend to these patients (Table 3). As individual histories can differ significantly, the treating pediatric surgeon should ensure a structured transfer into a specialized team in order to pass on all relevant information of the particular case.

### Biliary atresia and congenital choledochal malformations

Biliary atresia (BA) is a rare obliterative cholangiopathy affecting about one in 10,000 newborns in the Western World [39]. The standard primary treatment is the Kasai procedure that aims to establish bile flow in order to delay terminal liver damage. While most patients survive until adulthood the majority requires a liver transplantation during childhood or adolescence [40].

Up to 23% of BA patients are alive with native liver 20 years post-Kasai but struggle with long-term morbidities of liver disease such as portal hypertension or recurring cholangitis (Table 1) [41, 42]. HrQoL in patients with BA and native-liver survival is decreased compared to healthy individuals, but similar to patients post liver-transplant [41, 43]. Specific tools to measure HrQoL in BA patients mostly target transplanted patients while BA-specific instruments for survivors with native liver are lacking (Table 2).

A lifelong hepatological follow-up is essential for both transplanted patients and patients with native liver. Late adolescence is one of the most critical timepoints in patients post-transplant as this period often comes with reduced compliance regarding immunosuppressive therapy and consequently a risk of transplant rejection [44]. Overall, pediatric liver disease and its long-term problems are similar to adult hepatology, making this transition easy regarding adult practitioners’ expertise on disease-specific problems and challenges [45].

While specific transition programs for pediatric surgical hepatobiliary conditions are scarce, well-structured programs for pediatric liver diseases exist that generally include patients with history of BA [44], [45], [46], [47]. However, if necessary the pediatric surgeon should take responsibility to reach out to these highly specialized teams in order to ensure a smooth transition (Table 3).

Congenital choledochal malformations (CM) are congenital abnormal dilatations of the biliary tract occurring with an estimated incidence of one in 100,000 births in Western populations [48]. Surgical treatment consists of resection of the bile duct cysts but an increased risk to develop cholangiocarcinoma persists even after successful surgery [49]. While CDM patients generally do not suffer from hepatological or other long-term problems, a life-long follow-up is needed to screen for signs of malignancy [50]. The pediatric surgeon is responsible for communicating this to the patients as well as adult practitioners taking over the follow-up care during the transition period (Table 3).

### Congenital diaphragmatic hernia

Congenital diaphragmatic hernia (CDH) is a birth defect with an incidence of 2.3–3.6 per 10,000 livebirths [51]. An incomplete closure of the diaphragm causes prenatal herniation of abdominal organs into the thoracic cavity leading to subsequent severe pulmonary hypertension and lung hypoplasia in the newborn. After stabilization of the newborn patient, repositioning of the herniated organs and closure of the defect is performed via laparotomy or thoracoscopic approach. Long-term morbidities of CDH patients include impaired pulmonary function and neurological development as well as musculoskeletal changes and gastrointestinal sequelae such as gastroesophageal reflux disease or malnutrition [52, 53]. Evaluation of HrQoL in patients with CDH yields contradictory results. Condition specific instruments are lacking and methodological weaknesses of the studies available impede objective conclusions (Table 2) [54], [55], [56]. Overall, the existing data suggests a fairly equivalent HrQoL when comparing patients to the healthy population [57]. However, as patients suffer relevant long-term sequelae including severe respiratory and cardiological symptoms that can exacerbate as well as the frequent coexistence of concomitant anomalies a structured follow-up including a well-planned transition program is essential for CDH survivors (Table 3).

## Discussion

Many pediatric surgical patients suffer from long-term morbidities with a major impact on their HrQoL. These complex patients require life-long follow-up care of specialized physicians. The need for transitional medicine for pediatric surgical patients is evident and awareness has increased over recent years [58, 59]. However, implementation of systematic transition programs remains a challenge. While pediatric transitional care is improving, well-structured approaches for patient with pediatric surgical conditions are still scarce [60]. The little data available suggests a significant benefit of patients subjected to an organized transition program [22].

**Figure 2:**
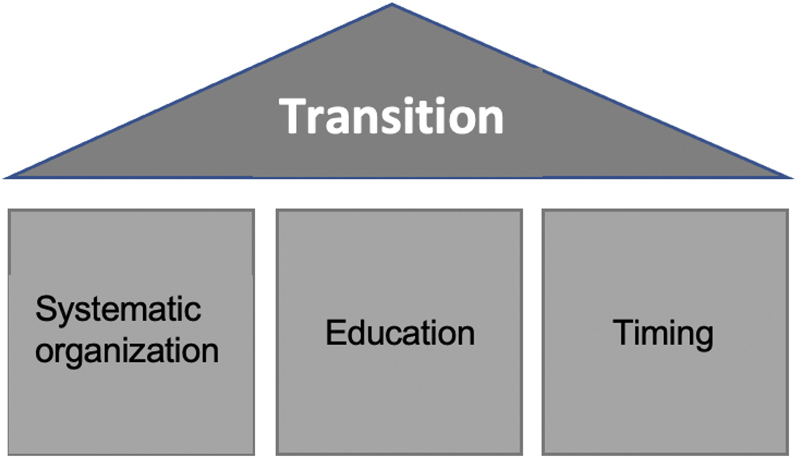
Key points of transition.

Key elements of a well-organized transition include a systematic approach with standardized schedules and treatment plans, thorough education of both the patient and the adult practitioner taking over the care as well as a careful choice of time period (Figure 2). HrQoL has been increasingly utilized as an outcome in health intervention and effectiveness studies and thus remains a major point of interest of long-term follow-up. The transition team should aim at working synergistically to ensure the patient’s individual needs and demands are met in order to provide optimal care and minimize negative impact on HrQoL during the transition period and beyond.

Most transition programs are centered on the last years of adolescence and first years of adulthood (16–21 years) [22, 46]. However, the most important aspect when considering an adequate timeframe is the mental development of each individual patient. In order to guarantee an effective transition, patients should have achieved an independent understanding of their disease as well as a certain degree of autonomy [61]. This provides the base for a successful transition into adult medicine where patients are self-responsible and no longer reliant on parents or guardians to help voice their needs and concerns.

Physicians should aim to provide a systematic approach to transition. A thorough and reliable documentation is essential to ensure no information is lost in the process. Standardized scheduling and therapeutic plans set in the beginning of the transition period provide structure for patients and family members during this challenging phase. Organization may be facilitated by engaging transition coordinators or case managers to oversee and direct this critical period. A successful continuous care of these complex patients can best be ensured in large centers offering the entire spectrum of specialized care including all diagnostic and therapeutic options needed to address the various long-term issues faced by pediatric surgical patients. While patients can often rely on family members and patient support groups to offer assistance and advice, asking patients to build their own outpatient network to meet their different needs is in our opinion not adequate and will most likely lead to loss of patients to regular follow-up.

The pediatric surgeon should be actively involved in the transition period to provide relevant details of the individual’s history and most importantly offer expertise regarding the management of the disease and its long-term morbidities. Disease-specific knowledge is essential to convey to adult-oriented caretakers that often lack specialized knowledge of these rare and complex diseases and their most relevant long-term problems. Educating both the patient as well as the adult practitioners taking over the patient’s care is one of the main responsibilities of the pediatric surgeon in transition medicine. If required, the pediatric surgeon should stay available to support patients and adult specialists even beyond the transition period.

The use of PROMs can assist the clinicians in the transition process as it provides a specific focus on the patient’s perspective. PROMs will help provide patient-centered data and in turn may stimulate self-management through involvement of the patient [62]. PROMs are increasingly used by pediatric surgeons and special attention should be paid to the patients’ HrQoL when evaluating outcomes and designing treatment strategies during transition. For pediatric surgeons and adult practitioners taking over the care, it is important that PROMs are selected carefully, to ensure they realize their full potential in playing a direct role in assisting the transition process. It is crucial that the instruments capture the aspects of health that matter the most to adolescents and young adults. The preferred approach is to include both generic and specific HrQoL instruments in order to provide a comprehensive and detailed evaluation of the patient’s perspective. However, this may be difficult to realize for conditions where specific instruments are not available (Table 2). In order to provide optimal care for these patients it is essential to not only implement well-structured transition programs but also strive to develop additional disease-specific PROMs for pediatric surgical conditions in order to allow for precise and reliable HrQoL assessment throughout the process of transition and beyond.

## Conclusions

The various conditions treated by pediatric surgeons often come with relevant long-term morbidities that require life-long medical care. Postoperative long-term care is one of the core-requirements of the modern pediatric surgeon who is not only responsible for the initial corrective surgery but should also attend to the critical phase of transition when patients are handed over to adult specialists. Multi-disciplinary approaches are needed to ensure optimal care for these complex patients in adult medicine. Careful selection of PROMs and development of disease- and/or treatment specific HrQoL instruments are essential to provide consistent objective outcome analysis during the transition process.

Systematic and well-structured transition programs including regular use of suitable PROMs need to be developed and implemented to reduce chronic symptoms and improve HrQoL in pediatric surgical patients.

## Supplementary Material

Supplementary Material Details
