# Supplementary material for: Need for transition medicine in pediatric surgery – health related quality of life in adolescents and young adults with congenital malformations
Source: Innov Surg Sci. 2022 Jan 7;6(4):151–60. doi: 10.1515/iss-2021-0019 (PMC9294337; doi:10.1515/iss-2021-0019)
Supplement: Supplementary file 1 — Supplementary Material Details [file j_iss-2021-0019_suppl.pdf]

Reviewer Assessment

M. Uecker et al.: Need for Transition Medicine in Pediatric Surgery – Health related Quality of Life in adolescents and young adults with congenital malformations

Reviewers' Comments to Original Submission

Reviewer 1: Midrio, Paola

Date received: 11-May-2021
Reviewer recommendation: **Return to author for minor modifications**
Reviewer overall scoring: **High**

Assessment Form scores: 5 = High/Yes; 3 = Medium/Adequate; 1 = Low

Is the subject area appropriate for the journal				2	
Does the title clearly reflect the paper's content?	5				
Does the abstract clearly reflect the paper's content	5				
Do the keywords clearly reflect the paper's content?		4			
Does the introduction present the problem clearly?	5				
Are the results/ conclusions justified?	5				
How comprehensive and up-to-date is the subject matter presented?	5				
How adequate is the data presentation?	5				
Are units and terminology used correctly?	5				
Is the number of cases adequate?		4			
Are the experimental methods/ clinical studies adequate?					
Is the length appropriate in relation to the content?		4			
Does the reader get new insights from the article?			3		
Please rate the practical significance.		4			
Please rate the accuracy of methods.		4			
Please rate the statistical evaluation and quality control.					
Please rate the appropriateness of the figures and tables.			3		
Please rate the appropriateness of the references.	5				
Please evaluate the writing style and use of language.	5				
Please judge the overall scientific quality of the manuscript.			3		
Are the methods used worthy of reproduction in greater deal?	Yes				
Would you be willing to review a revision of this manuscript?	Yes				

Comments to author: The paper is well written and addresses in an exhaustive way the complex aspects of transition of care of children treated for congenital malformation who grow up and face the adult world.
Table 2 is redundant.
The reference list quite long and some papers can be grouped together.

Reviewer 2: Tekant, Gonca

Date received: 06-Jun-2021
Reviewer recommendation: **Accept in present form**
Reviewer overall scoring: **Excellent**

Assessment Form scores: 5 = High/Yes; 3 = Medium/Adequate; 1 = Low

Is the subject area appropriate for the journal	5				
Does the title clearly reflect the paper's content?	5				
Does the abstract clearly reflect the paper's content	5				
Do the keywords clearly reflect the paper's content?	5				
Does the introduction present the problem clearly?	5				
Are the results/ conclusions justified?	5				
How comprehensive and up-to-date is the subject matter presented?	5				
How adequate is the data presentation?	5				
Are units and terminology used correctly?	5				
Is the number of cases adequate?	5				
Are the experimental methods/ clinical studies adequate?	5				
Is the length appropriate in relation to the content?	5				
Does the reader get new insights from the article?	5				
Please rate the practical significance.	5				
Please rate the accuracy of methods.	5				
Please rate the statistical evaluation and quality control.	5				
Please rate the appropriateness of the figures and tables.	5				
Please rate the appropriateness of the references.	5				
Please evaluate the writing style and use of language.	5				
Please judge the overall scientific quality of the manuscript.	5				
Are the methods used worthy of reproduction in greater deal?	Yes				
Would you be willing to review a revision of this manuscript?	Yes				

Comments to author: Thank you for inviting me to evaluate the manuscript titled 'Need for Transition Medicine in Pediatric Surgery – Health related Quality of Life in adolescents and young adults with congenital malformations'.
This review article adresses all the problems encountered in patients with congenital malformations and health related quality of life measures in adolescents and young adults.
I believe the manuscript is suitable for publication in Innovative Surgical Sciences in its present form.

Authors' Response to Reviewer Comments

Date received: 16-Jun-2021

Response to reviewer 1

Thank you very much for your review and comments regarding our manuscript.

- *In our opinion Table 2 is essential as it provides an overview of available instruments to assess HrQoL for pediatric surgical conditions at a quick glance. Furthermore, it highlights the lack of disease-specific items for some malformations. As both of these elements are discussed in our manuscript, we would like to keep the table in order to summarize and underline our points regarding HrQoL evaluation for pediatric surgical patients.*
- *We have adjusted the reference list and grouped together some of the papers in order to shorten it.*

Response to reviewer 2

Thank you very much for your review and positive comments regarding our manuscript

Comment by the Guest Editor of the Thematic Issue on the revised version

All reviewer comments were addressed adequately and the manuscript should be published in its present stage.
